# Comparison of continence outcomes of early catheter removal on postoperative day 2 and 4 after laparoscopic radical prostatectomy: a randomized controlled trial

**DOI:** 10.1186/s12894-015-0065-y

**Published:** 2015-07-31

**Authors:** Masashi Matsushima, Akira Miyajima, Seiya Hattori, Toshikazu Takeda, Ryuichi Mizuno, Eiji Kikuchi, Mototsugu Oya

**Affiliations:** Department of Urology, Keio University School of Medicine, 35 Shinanomachi, Shinjuku-ku, Tokyo, 160-8582 Japan

**Keywords:** Laparoscopic radical prostatectomy, Early catheter removal, Urinary incontinence, Prostate cancer

## Abstract

**Background:**

The optimal timing of catheter removal following laparoscopic radical prostatectomy (LRP) has not yet been determined. This prospective study was designed to compare the efficacy and safety of catheter removal on postoperative day (POD) 2 versus POD 4 after LRP and its impact on urinary continence outcomes.

**Methods:**

One hundred and thirteen patients underwent LRP and were prospectively randomized into two groups: group 1 (*n* = 57) had the urinary catheter removed on POD 2 while group 2 (*n* = 56) had the catheter removed on POD 4. The urine loss ratio (ULR) was defined as the weight of urine loss in the pad divided by the daily micturition volume. Continence was defined as a pad-free status.

**Results:**

No significant differences were observed in clinical features between groups 1 and 2. Acute urinary retention (AUR) after catheter removal occurred in 21 patients (18.6 %) (13 (22.8 %) in group 1 and 8 (14.3 %) in group 2 (*p* = 0.244). The first-day mean ULR values were 1.16 ± 4.95 in group 1 and 1.02 ± 3.27 in group 2 (*p* = 0.870). The last-day mean ULR values were 0.57 ± 1.60 in group 1 and 2.78 ± 15.49 in group 2 (*p* = 0.353). Continence rates at 3, 6, 9, and 12 months were 21.8, 41.1, 58.0, and 71.4 % in group 1 and 34.5, 66.0, 79.2, and 83.7 % in group 2 (*p* = 0.138, 0.009, 0.024, and 0.146, respectively). In AUR cases, continence rates at 3, 6, 9, and 12 months were 0, 23.1, 38.5, and 54.5 % in group 1 and 37.5, 75.0, 87.5, and 87.5 % in group 2 (*p* = 0.017, 0.020, 0.027, and 0.127, respectively). A multivariate analysis identified AUR after catheter removal on POD 2 as the only predictive factor for incontinence 6 and 9 months after LRP (*p* = 0.030 and 0.018, respectively).

**Conclusions:**

Our results demonstrated that early catheter removal on POD 2 after LRP may increase the risk of incontinence.

**Trial registration:**

The study was registered as Clinical trial: (UMIN000014944); registration date: 12 March 2012.

## Background

The management of patients after radical prostatectomy (RP) has historically been associated with a long period of catheterization to allow anastomotic healing. Traditionally, the duration of catheterization has averaged from 10 to 21 days at most urologic centers [1–3]. However, there is currently no objective evidence to support the use of indwelling urinary catheters for such long periods after RP [4]. Furthermore, previous studies on the feasibility of early catheter removal after RP have reported a low complication rate with a high rate of successful catheter removal [5–7].

Several studies reported that protracted catheterization was a major source of discomfort and irritation in patients after RP [4, 5]. Therefore, the indwelling urinary catheter needs to be removed as early as possible without jeopardizing the outcome. A recent study demonstrated that it was safe to remove catheters in most patients 3 to 4 days after RP if cystography showed no urinary extravasation [7].

The technique of laparoscopic radical prostatectomy (LRP) has gained worldwide acceptance as a treatment for localized prostate cancer since the first feasibility report by Schuessler et al. in 1997 and standardization of the technique by Guillonneau et al. in 1999 [8, 9]. The advantages of LRP have been supported by multiple studies and include a shorter inpatient stay, better pain control, faster return to everyday activities, and decreased short-term complications [10]. A definite advantage may be reduced catheterization time after LRP because vesicourethral anastomosis (VUA) is performed under direct vision, and there is better luminosity and magnification with no blind knotting [11]. Nadu et al. revealed the absence of contrast medium leakage in 84.9 % of patients 2-4 days after LRP, and also that urethral catheter removal could then be safely performed [11].

Urinary incontinence is one of the most feared complications of RP, LRP, and robot-assisted LRP (RALP). In a recent meta-analysis, continence rates 12 months after LRP ranged from 66 % to 95 % [12]. The time to continence after removal of a urinary catheter is a common clinical question. Therefore, the urine loss ratio (ULR) after catheter removal has been suggested as a reliable measure to predict the severity and duration of urinary incontinence [13–15].

To the best of our knowledge, the effects of the differential timing of early catheter removal have not yet been elucidated in detail. Therefore, the aim of the present study was to compare the efficacy and safety of urinary catheter removal on postoperative day (POD) 2 versus POD 4 after LRP and its impact on urinary continence outcomes.

## Methods

**Fig. 1:**
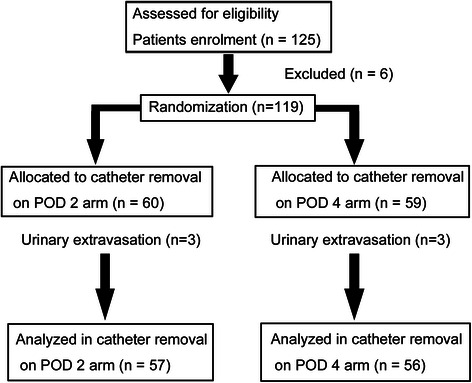
Study consort diagram. POD, postoperative day

Between March 2012 and September 2014, 125 patients with clinically localized prostate cancer underwent LRP performed by the same experienced surgeon (≥300 LRP cases at study initiation) at Keio University Hospital. Inclusion criteria were as follows: localized prostate cancer without lymph node and distant metastasis and age <75 years. Exclusion criteria were as follows: previous radiotherapy; previous prostatic, bladder neck, urethral, or pelvic surgery; and the presence of an indwelling urinary catheter (*N* = 6). The remaining 119 patients were randomly divided into two groups (1:1) before surgery on the basis of the timing of their catheter removal after LRP. Randomization was carried out after consent using a computer generated random table by an independent researcher who was not directly involved with the study. Random blocks of different lengths were used. Group 1 had the urinary catheter removed on POD 2, while group 2 had the catheter removed on POD 4. Blinding was not possible in this trial because the timing of catheter removal was different. Cystography revealed leakage in three patients in group 1 (5.3 %) on POD 2 and in three patients in group 2 (5.4 %) on POD 4. These six patients with extravasation were excluded from the data analysis. We identified 113 patients who were followed-up for at least 3 months after LRP as our prospective study population. The primary end-point of this study was the continence rate, and secondary end-points were other complications. A study consort diagram of the randomization procedure is given in Fig. 1.

Ethical approval for the design of this study was granted by the Keio University Hospital Ethical Committee. Prior to undergoing surgery, all patients were informed of the objectives of the present study as well as the timing of catheter removal after LRP (POD 2 or POD 4). Written informed consent was obtained from all patients prior to participation in this study. This study was registered with the University Hospital Medical Information Network Clinical Trials Registry in Japan (UMIN000014944) on 12 March 2012.

### Surgical technique

LRP was performed under general anesthesia using an extraperitoneal 5-port approach. Insufflation pressure was typically maintained at 10 mmHg during surgery. Bilateral dissection was limited to the lymph nodes along the external iliac vein and in the obturator fossa. Posterior reconstruction of the rhabdosphincter was performed before VUA, as described by Rocco et al. [16]. V-Loc 180 (barbed polyglyconate suture; Covidien, Mansfield, USA) was used for VUA, and a 20-Fr Foley catheter was inserted after VUA. The integrity of the anastomosis was tested intraoperatively by instilling 120 mL of saline into the bladder. At the end of surgery, an 8-mm drainage tube was placed in the prevesical space. The tube was removed when drainage was less than 100 mL per day, which in many cases was on POD 1.

Gravitational cystography was performed on POD 2 or 4 after LRP to check VUA. During cystography, the catheter was advanced slightly to prevent it from compressing the bladder neck, and the bladder was filled with 140 mL of contrast media. Removal of the urinary catheter was performed when the cystogram was normal without any urinary extravasation. If any extravasation of contrast media was observed, the catheter was left in place, and cystography was repeated after a few days. If urinary retention was noted, the catheter was reinserted.

After catheter removal, the 24-h pad test was performed each day during the remaining hospital stay. The 24 hourly total micturition volume was also measured every day until discharge. ULR was defined as the weight of urine loss in the pad divided by the daily micturition volume, distinguishing between ULR on the first day after catheter removal and on the last day of the hospital stay. Previous studies reported that ULR predicted the time to continence [13–15]. The first-day ULR was defined as ULR on the day of catheter removal, and the last-day ULR was described as ULR on the last day of the hospital stay. The maximum ULR was defined as the maximum ULR during the hospital stay, while the minimum ULR was defined as the minimum ULR during the hospital stay. Patients did not take any therapeutic agents for urinary incontinence during the measurement of ULR.

During the follow-up, patients were asked how many pads they required daily. ULR during hospitalization and the number of pads 1, 3, 6, 9, and 12 months postoperatively were analyzed. Urinary continence was defined as a pad-free status. Complications and continence during the immediate and late postoperative periods were assessed during a follow-up period that ranged from 3 to 30 months (mean 19.9 ± 7.0 months). The continence outcomes of men whose catheters were removed on POD 2 were compared with the outcomes of those whose catheters were removed on POD 4.

### Statistical analysis

The power calculation for this study was based on the primary end-point of urinary continence. The minimum clinically important difference was estimated to be 25 %, based on our clinical judgment because no previous similar study has provided continence rates following early catheter removal on different days after LRP. A sample size of 43 patients per arm was required to provide a power of 80 % in order to detect a difference of 25 % with a 2-sided alpha error of 0.05, and adjusting by 20 % for potential dropouts gave a final sample size of 113.

All values are presented as the mean ± standard deviation (S.D.). The Student’s *t*-test and Mann–Whitney *U* test were used to assess quantitative parametric and nonparametric variables, respectively. The chi-square test was used to assess differences in distributions between categorical parameters. A logistic regression analysis was used to identify a significant set of independent predictors of incontinence 6 and 9 months after LRP. Significance was determined as *p* < 0.05. All analyses were performed using IBM® SPSS® Statistics Version 21 (International Business Machines Corporation, New York, USA).

## Results

All LRP procedures were performed safely with no serious complications and no open conversion. There was no intraoperative urinary leakage. The mean age of patients was 65.9 ± 5.5 years, the mean preoperative PSA level was 9.0 ± 6.7 ng/mL, and the median follow-up interval was 21 (3–30) months. The mean prostate volume was 30.2 ± 11.3 mL. The clinical stage was T1c in 38 patients, T2a in 53, T2b in 4, and T2c in 18. The biopsy Gleason score was ≤6 in 22 patients, 7 in 73, and ≥8 in 18. The mean operative time was 177.2 ± 37.4 min, including lymph node dissection. Average blood loss, including urine volume, was 208.2 ± 246.9 mL. Table 1 summarizes the characteristics of the patient population, including age, PSA, prostate volume, biopsy Gleason score, clinical T stage, presence of nerve sparing, operative time, and blood loss.

**Table 1:** Clinical characteristics of patients who underwent LRP

		No of Pts	%
Age	<65	37	32.7
	≥65	76	67.3
PSA before LRP	<10	83	73.5
	≥10	30	26.5
Prostate volume (mL)	<30	66	58.4
	≥30	47	41.6
Biopsy Gleason score	≤6	22	19.5
	7	73	64.6
	≥8	18	15.9
Clinical T stage	T1c	38	33.6
	T2a	53	46.9
	T2b	4	3.5
	T2c	18	15.9
Nerve sparing	+	26	23.0
	−	87	77.0
Operative time (min)	<150	32	28.3
	≥150	81	71.7
Blood loss (mL)	<100	42	37.2
	≥100	71	62.8
Total cases		113	

*LRP* laparoscopic radical prostatectomy, *Pts* patients

No significant differences were observed in clinical characteristics between groups 1 and 2 (Table 2). Acute urinary retention (AUR) after catheter removal occurred in 21 patients (18.6 %) (13 (22.8 %) in group 1 and 8 (14.3 %) in group 2 (*p* = 0.244)). These patients were treated with simple catheter replacement for a few days. In every case, the catheter was replaced easily without cystoscopy or fluoroscopy. None of the AUR patients developed hematuria or clots. Bladder neck contracture was not observed.

**Table 2:** Comparison of clinical characteristics between group 1 (catheter removal on POD 2) and group 2 (catheter removal on POD 4)

		Group 1 (*n* = 57)	Group 2 (*n* = 56)	*p* value
Age	<65	16	21	0.286
	≥65	41	35	
PSA before LRP	<10	46	37	0.078
	≥10	11	19	
Prostate volume	<30	33	33	0.911
	≥30	24	23	
Gleason score	≤6	10	12	0.602
	≥7	47	44	
Clinical T stage	T1c	16	22	0.207
	T2a,b,c	41	34	
Nerve sparing	+	13	13	0.959
	−	44	43	
Operative time	<150	19	13	0.233
	≥150	38	43	
Blood loss	<100	24	18	0.273
	≥100	33	38	
AUR	+	13	8	0.244
	−	44	48	

*POD* postoperative day, *LRP* laparoscopic radical prostatectomy, *AUR* acute urinary retention

The first-day mean ULR values were 1.16 ± 4.95 in group 1 and 1.02 ± 3.27 in group 2 (*p* = 0.870). The last-day mean ULR values were 0.57 ± 1.60 in group 1 and 2.78 ± 15.49 in group 2 (*p* = 0.353). The maximum mean ULR values were 1.48 ± 5.13 in group 1 and 2.93 ± 15.47 in group 2 (*p* = 0.558). The minimum mean ULR values were 0.22 ± 0.35 in group 1 and 0.85 ± 3.24 in group 2 (*p* = 0.206). No significant differences were observed between the two groups (Table 3).

**Table 3:** Comparison of ULR and continence rates between group 1 (catheter removal on POD 2) and group 2 (catheter removal on POD 4)

		Group 1 (*n* = 57)	Group 2 (*n* = 56)	*p* value
ULR	First-day mean ULR	1.16 ± 4.95	1.02 ± 3.27	0.870
	Last-day mean ULR	0.57 ± 1.60	2.78 ± 15.49	0.353
	Maximum mean ULR	1.48 ± 5.13	2.93 ± 15.47	0.558
	Minimum mean ULR	0.22 ± 0.35	0.85 ± 3.24	0.206
Continence				
1 month after LRP	+	2 (3.6 %)	3 (5.4 %)	0.647
	-	54 (96.4 %)	53 (94.6 %)	
3 months after LRP	+	12 (21.8 %)	19 (34.5 %)	0.138
	-	43 (78.2 %)	36 (65.5 %)	
6 months after LRP	+	23 (41.1 %)	35 (66.0 %)	0.009
	-	33 (58.9 %)	18 (34.0 %)	
9 months after LRP	+	29 (58.0 %)	38 (79.2 %)	0.024
	-	21 (42.0 %)	10 (20.8 %)	
12 months after LRP	+	35 (71.4 %)	41 (83.7 %)	0.146
	-	14 (28.6 %)	8 (16.3 %)	

*POD* postoperative day, *ULR* urine loss ratio, *LRP* laparoscopic radical prostatectomy

Continence rates 3, 6, 9, and 12 months after removal of the urinary catheter were 21.8, 41.1, 58.0, and 71.4 % in group 1 and 34.5, 66.0, 79.2, and 83.7 % in group 2 (*p* = 0.138, 0.009, 0.024, and 0.146, respectively) (Table 3). Continence rates 6 and 9 months after LRP were significantly lower in group 1 than in group 2. However, if patients with AUR were excluded from this analysis, these differences became insignificant.

In AUR cases, continence rates 3, 6, 9, and 12 months after removal of the urinary catheter were 0, 23.1, 38.5, and 54.5 % in group 1 and 37.5, 75.0, 87.5, and 87.5 % in group 2 (*p* = 0.017, 0.020, 0.027, and 0.127, respectively) (Table 4). In patients with AUR, continence rates 3, 6, and 9 months after LRP were significantly lower in group 1 than in group 2.

**Table 4:** Comparison of continence rates in AUR cases between group 1 (catheter removal on POD 2) and group 2 (catheter removal on POD 4)

	Continence	AUR cases in group 1 (*n* = 13)	AUR cases in group 2 (*n* = 8)	*p* value
1 month after LRP	+	0 (0 %)	0 (0 %)	-
	-	13 (100 %)	8 (100 %)	
3 months after LRP	+	0 (0 %)	3 (37.5 %)	0.017
	-	13 (100 %)	5 (62.5 %)	
6 months after LRP	+	3 (23.1 %)	6 (75 %)	0.020
	-	10 (76.9 %)	2 (25 %)	
9 months after LRP	+	5 (38.5 %)	7 (87.5 %)	0.027
	-	8 (61.5 %)	1 (12.5 %)	
12 months after LRP	+	6 (54.5 %)	7 (87.5 %)	0.127
	-	5 (45.5 %)	1 (12.5 %)	

*AUR* acute urinary retention, *POD* postoperative day, *LRP* laparoscopic radical prostatectomy

A multivariate analysis (Table 5) identified AUR after catheter removal on POD 2 as the only independent predictor of incontinence 6 months after LRP (odds ratio, 4.472; *p* = 0.030). Age, PSA, prostate volume, the Gleason score, clinical stage, nerve sparing, operative time, blood loss, or AUR after catheter removal on POD 4 had no effect on the continence rate 6 months after LRP. Similar results were observed in the multivariate analysis of factors affecting incontinence 9 months after LRP (odds ratio, 4.313; *p* = 0.018).

**Table 5:** Analysis of factors affecting incontinence 6 months after LRP

	Univariate analysis (*p* value)	Multivariate analysis (*p* value)	Standard error	Odds ratio
Age <65 vs. ≥65	0.949			
PSA before LRP <10 vs. ≥10	0.851			
Prostate volume <30 vs. ≥30	0.566			
Gleason score ≤6 vs. ≥7	0.688			
Clinical T stage T1c vs. T2a,b,c	0.623			
Nerve sparing yes vs. no	0.293			
Operative time <150 vs. ≥150	0.203			
Blood loss <100 vs. ≥100	0.264			
AUR on POD 2 yes vs. no	0.020	0.030	0.690	4.472
AUR on POD 4 yes vs. no	0.200			

*LRP* laparoscopic radical prostatectomy, *AUR* acute urinary retention, *POD* postoperative day

## Discussion

This prospective study was designed to compare the efficacy and safety of catheter removal on POD 2 versus POD 4 after LRP and its impact on urinary continence outcomes. In this study, 94.7 % of men undergoing cystography on POD 2 or 4 exhibited no evidence of urinary extravasation. The main complication associated with early catheter removal in this study was AUR. A total of 18.6 % of men who had catheters removed on POD 2 or 4 developed AUR. Although no significant differences were observed between the two groups in terms of clinical characteristics, AUR rate, or average ULR (first-day, last-day, maximum, or minimum), continence rates 6 and 9 months after LRP were significantly lower in group 1 (POD 2) than in group 2 (POD 4). In AUR cases, continence rates 3, 6, and 9 months after LRP were significantly lower in group 1 than in group 2. Moreover, a multivariate analysis identified AUR after catheter removal on POD 2 as the only predictive factor for incontinence 6 and 9 months after LRP. Meanwhile, AUR after catheter removal on POD 4 had no effect on the continence rate. Therefore, we consider it premature to remove the urinary catheter on POD 2 following LRP with a running VUA. To the best of our knowledge, our prospective study is the first to identify a relationship between the risk of incontinence and AUR following earlier catheter removal.

The duration of indwelling catheter use after RP, LRP, and RALP has progressively shortened; however, the optimal timing of removal has not yet been determined. Traditionally, urinary catheter removal after RP has been performed between 10 and 21 days postoperatively without any evidence [1–3]. However, some centers remove the urinary catheter between 5 and 12 days after RALP [17–19]. The advantages of early catheter removal include improved quality of life (QOL) and lower infection rate and bladder irritability symptoms [4, 11]. In one study, the majority of men who underwent RP indicated that the urinary catheter was more of a concern than postoperative pain [4]. Conversely, proponents of longer catheterization claim that early removal is associated with a risk of urinary extravasation, which, in turn, may lead to pelvic abscess, urinoma, urinary incontinence, or anastomotic stricture [20, 21]. AUR following urinary catheter removal on POD 2 was identified as the only predictive factor for incontinence after LRP in our study. However, other studies demonstrated that early catheter removal was associated with a significantly higher continence rate after RP and LRP [22, 23]. In these studies, early catheter removal was defined as catheter removal on POD 4 or on or before POD 7, and no patients had catheters removed on POD 2. In the present study, all patients underwent early (POD 2 or POD 4) catheter removal, and any patient who underwent late catheter removal, such as after POD 7, was not included. However, to the best of our knowledge, no previous studies have compared the continence outcomes of urinary catheter removal on POD 2 with those of catheter removal after POD 2, such as POD 4. Thus, the present study evaluated the effects of the differential timing of early catheter removal.

Although catheter drainage to prevent urinary extravasation may reduce the risk of urinary incontinence, it is equally plausible that prolonged catheterization may contribute to urinary incontinence secondary to mechanical damage and inflammation of the urethral and bladder mucosa [22]. It is important to note that AUR and reinsertion of a catheter following earlier catheter removal (e.g., on POD 2) may have increased the risk of urinary incontinence by urinary extravasation and mechanical damage of the urethra. In our prospective study, AUR on POD 2 after catheter removal was the only predictive factor for incontinence after LRP. Therefore, our hypothesis that early catheter removal (≤7 days after surgery) is associated with good continence held true; however, POD 2 may be premature for catheter removal because of the risk of incontinence with AUR.

The evolution of minimally invasive techniques for the treatment of prostate cancer, such as LRP and RALP, has reduced postoperative pain and the duration of catheterization [11, 18, 24]. These improvements have been attributed to the development of intracorporeal suturing techniques with visualization of VUA. These technical advances have allowed us to challenge previous postoperative management plans. Removal of the urinary catheter on POD 4 has become routine in centers offering LRP [7, 11, 23]. Nadu et al. investigated a series of LRP cases using cystography and demonstrated that early urinary catheter removal (POD 2 and POD 4) was possible [11]. Eighty-five percent of men in that study exhibited no evidence of extravasation, and urinary catheters were successfully removed. This high success rate was attributed to the superior anastomosis achieved laparoscopically. However, that study did not include a subgroup analysis of urinary catheter removal on POD 2. The results of the present study showed that early catheter removal on POD 2 after LRP with a running VUA may increase the risk of incontinence.

AUR appears to be a risk after LRP and occurred in 21 (18.6 %) patients without clot formation in our series. Although all patients with AUR had their catheters reinserted without complications, a severe impact on continence was observed in group 1 (catheter removed on POD 2). The etiology of AUR after LRP is likely to be postoperative anastomotic edema, postoperative pain, or increased tone of the bladder neck smooth muscle [25]. Normal micturition is always re-established after several days of catheterization. Therefore, one explanation for the high rate of AUR with early catheter removal may be the presence of anastomotic edema. The incidence of AUR after early catheter removal was previously reported to be between 6.7 and 21.0 % [11, 25, 26].

Several studies have specifically investigated the incidence of anastomotic stricture after RP without early catheter removal, and reported rates ranging between 4.8 and 15 % [27–29]. Previous transurethral resection of the prostate, a history of smoking, and urinary extravasation have been associated with an increased rate of anastomotic stricture [28, 29]. Koch et al. showed that early catheter removal did not increase the incidence of anastomotic stricture over that of historical controls or previously reported rates [5]. In the present study, no patient had anastomotic stricture, which was better than previously reported findings. Therefore, the results of our study suggest that catheter removal on POD 2 or 4 did not promote stricture formation.

Urinary incontinence after RP has a significant impact on QOL and continues to be a major concern for patients [5]. Reported continence rates 1 year postoperatively ranged from 60 to 93 % after RP, from 66 to 95 % after LRP, and from 69 to 97 % after RARP [12, 30]. By defining continence as a pad-free status, the continence rate in the present study at 12 months was 83.7 % in group 2, which was within the average range [12]. Patients who had catheters removed on POD 4 showed the normal recovery of urinary continence.

Our study has a number of limitations. The overall sample size was small. We relied on patient reports of the degree of continence and use of protective pads. Therefore, urinary continence was not assessed using an objective test. Despite these limitations, we believe that our results indicate that early catheter removal on POD 2 increases the risk of incontinence with AUR and needs to be avoided in clinical practice.

## Conclusions

In terms of the risk of urinary incontinence after LRP, in most patients, urinary catheter removal was safer on POD 4 than on POD 2.

## Abbreviations

LRP: Laparoscopic radical prostatectomy
POD: Postoperative day
ULR: The urine loss ratio
AUR: Acute urinary retention
RP: Radical prostatectomy
VUA: Vesicourethral anastomosis
RALP: Robot-assisted LRP
S.D.: Standard deviation
QOL: Quality of life
